# Cancer disease progression and death during the COVID-19 pandemic: a multidisciplinary analysis for the Peruvian setting

**DOI:** 10.3332/ecancer.2020.1098

**Published:** 2020-09-08

**Authors:** Juan Astigueta-Pérez, Milagros Abad-Licham, Carlos Chávez-Chirinos, Luis Beraun-Milla, Alberto Lachos-Dávila, Elizabeth Diaz-Pérez, Karem Portugal-Valdivia, Paul Pilco Castañeda, Isaías Pérez Alférez, Edward Mezones-Holguín

**Affiliations:** 1 Departamento de Cirugía Oncológica, Instituto Regional de Enfermedades Neoplásicas Norte, Trujillo 13600, Peru; 2 Facultad de Medicina, Universidad Privada Antenor Orrego, Trujillo 13007, Peru; 3 Centro de Excelencia en Patología Oncológica, Trujillo 13007, Peru; 4 Departamento de Patología Oncológica, Instituto Regional de Enfermedades Neoplásicas Norte, Trujillo 13600, Peru; 5 Departamento de Cirugía Oncológica, Instituto Regional de Enfermedades Neoplásicas Sur, Arequipa 04000, Peru; 6 Departamento de Cirugía Oncológica, Instituto Regional de Enfermedades Neoplásicas Centro, Huancayo 12000, Peru; 7 Departamento de Radioterapia, Instituto Nacional de Enfermedades Neoplásicas, Lima 15000, Peru; 8 Departamento de Especialidades Médicas, Instituto Nacional de Enfermedades Neoplásicas, Lima 15000, Peru; 9 Departamento de Oncología, Hospital Santa Rosa, Lima 15000, Peru; 10 Cirugía Oncológica, Clínica Delgado, Lima 15000, Peru; 11 Archivo Regional del Gobierno Regional de Tacna, Universidad Nacional Jorge Basadre, Tacna 23004, Peru; 12 Centro de Excelencia en Investigaciones Económicas y Sociales en Salud, Universidad San Ignacio de Loyola, Lima 15012, Peru; 13 Epi-Gnosis Solutions, Piura 20001, Peru; a https://orcid.org/0000-0001-5984-3270; b https://orcid.org/0000-0002-3530-6937; c https://orcid.org/0000-0002-5724-0684; d https://orcid.org/0000-0002-6190-1959; e https://orcid.org/0000-0001-7082-6930; f https://orcid.org/0000-0001-7168-8613; g https://orcid.org/0000-0002-4851-8664

**Keywords:** COVID-19, SARS-COV-2, pandemic, cancer, healthcare systems, Peru

## Abstract

Since the COVID-19 pandemic began in China in late 2019, infection from the SARS-CoV-2 virus has spread virtually worldwide. This infection has adversely affected several countries; governments have outlined a series of political measures aimed to preserve the health and safety of their populations. In Peru, most actions have prioritised COVID-19 attention, with a subsequent gap in the healthcare facilities needed for other diseases. Cancer, one principal cause of death in the country, is usually diagnosed late. Moreover, in the pandemic context, the prevention and control of cancer have been negatively affected. Therefore, we carried out a multidisciplinary analysis using the Ishikawa diagram to identify the probable factors that contribute to cancer progression and deaths in Peru.

## Introduction

The pandemic caused by SARS-COV-2 affects the countries with repercussions beyond their healthcare systems. Since the first case, reported in Wuhan, China, in late 2019, the virus has spread virtually worldwide. In January 2020, the World Health Organization (WHO) declared an international public health emergency [1]. The repercussions continue to grow due to mortality and morbidity directly related to this infection and also collateral effects associated with the mitigation measures implemented by governments [2]. The impact of COVID-19 is not limited to the health arena; this epidemic has also shaken economic structures and accentuated inequalities, particularly in low- and middle-income countries [3]. Similarly, there are emerging concerns that the prioritisation of COVID-19 in national agendas has detracted attention from other health needs, including cancer [4].

In this context, the Peruvian government has applied several measures to mitigate the epidemic. Although it had questionable adherence, the Peruvian lockdown which lasted more than 120 days was one of the longest and strictest in Latin America. During this time, most institutions in the Peruvian healthcare system (PHS) focused their efforts on COVID-19 [5]. This situation has meant a subsequent reduction in the range of health services available for other illnesses and health conditions. Oncology is one of the worst-affected areas in the health sector, where the epidemic has severely hampered early diagnosis and timely treatment, resulting in further progression of the disease and mortality in cancer patients [6]. The so-called ‘cancer crisis amid the pandemic’ [4] would also reach Peru. Nevertheless, to the best of the authors’ knowledge, no empirical evidence has been published in this regard.

The implementation of actions to control and manage cancer in developing countries is critical although these activities diminished during the COVID-19 pandemic [7]. Cancer is the second-highest cause of death in the world, with 9.6 million fatalities in 2018 and approximately 70% from low- and middle-income countries such as Peru [8–10]. Some initiatives advocate and promote the care of cancer patients, including from prevention to specialised therapeutic management; nonetheless, these actions have declined during the COVID-19 pandemic [11, 12]. In this health disaster framework, cancer patients and their families have become victims of collateral damage from the COVID-19 epidemic. Hence, the need for a comprehensive and objective evaluation of the situation in the country has emerged.

Therefore, we constituted a team of specialist from different healthcare and academic institutions in Peru to carry out a multidisciplinary approach that identifies potentially causal factors of disease progression and death from cancer. In this manuscript, first, we present relevant information about the dynamics of cancer in the complexity of the Peruvian Healthcare System; then, we describe the analysis performed, and finally, we offer some reflections.

## Background

The estimated increase in the cumulative incidence of cancer will be higher in low- and middle-income countries than high-income countries at 2030; furthermore, there is a substantial gap in achieving an equitable response amongst their healthcare systems [13, 14]. In Peru, cancer is the primary cause of death (2018: 92.9 cases per 100,000 inhabitants), and the highest mortality rates originate from the stomach, lungs, prostate, liver, bile ducts and colon. Cancer causes disability, quality-of-life impairment and potential years of life lost due to premature death [9, 10]. The incidence rate was 192.6 in 2018, with notable differences in the nosological presentation by sex in the past 5 years [8]. Hence, it is necessary to implement strategies to foster universal access to oncology services [15, 16]. This situation is more troubling in Peru, where the supply of specialised health services in oncology is still insufficient and most existing infrastructure is outdated, with hospitals ranging on average between 50 and 100 years old [17, 18]. Furthermore, another alarming issue is the meagre budget allocated to health; in 2020, it reached just 2.2% of Peruvian Gross Domestic Product, one of the lowest in Latin America [19]. The hospitals operated by the Ministry of Health (MINSA, from its Spanish acronym) and Regional Governments (GORE, from its Spanish acronym) do not meet the recommended international quality standards, and there are significant deficiencies in the resolution capacity at different levels; besides, these institutions deal with administrative problems and suffer from the severe shortages of medicines and medical devices [17].

**Figure 1.**
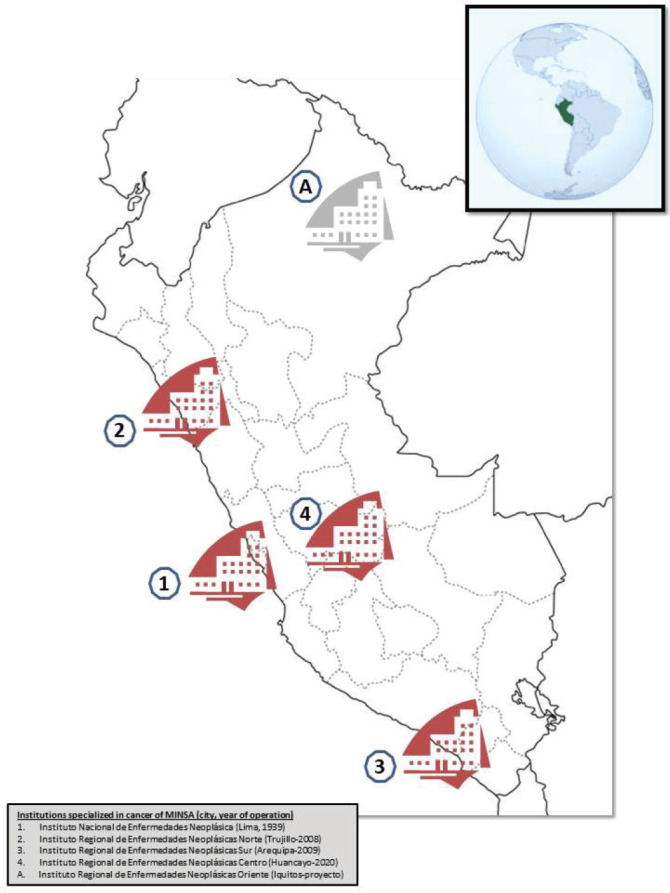
Distribution of specialised cancer institutes of the Ministry of Health in Peru.

Peru is moving towards universal health coverage although with limitations and deficiencies. The Peruvian Health System (PHS) is fragmented and segmented, with the public and private sectors involved in financing and healthcare provision [18, 20]. Since the Universal Health Insurance Act (AUS) in 2009, population coverage has reached 75% in 2017, slightly more than half in Comprehensive Health Insurance (SIS from its Spanish Acronym, mainly subsidised), and 22% in Social Security (EsSalud, mainly contributory); furthermore, around 70% of the population receives medical attention in MINSA and GORE facilities [21]. Nevertheless, there are significant problems with access to formal health services [22] and medication, especially in the public sector [23, 24]. Regarding cancer, MINSA has four specialised institutes: the National Cancer Institute (INEN, from its Spanish acronym) in the capital city (Lima, 1939) and three Regional Cancer Institutes (IREN, from the Spanish acronym) in northern (Trujillo, 2008), southern (Arequipa, 2009) and central (Huancayo, 2020). Furthermore, there are oncology units in 12 hospitals in Lima and seven in other regions [11, 12]. Similarly, there is a project for another IREN in Iquitos city to cover the northeastern in the jungle area (Figure 1).

Despite the particular characteristics of PHS and its boundaries, the Peruvian government has positioned cancer as a national health priority. In 2012, the President launched the ‘Plan Esperanza’ pursuing the reduction in cancer mortality and morbidity by providing comprehensive care and improving access to oncology services across the country [12]. The plan spurred the development of strategic oncology programmes that focus on a few cancers, such as cervical, breast, colon, stomach and prostate cancer, as well as leukaemia and lymphomas. Initiatives such as Plan Esperanza and others from non-governmental institutions have achieved progress in cancer management and control in the past 10 years. That headway, however, has been insufficient due to the chronic postponement of investment in the public health system [17, 18]. Beyond these efforts, official information from MINSA shows that more than 60% of new cases of cancer in 2013–2017 were detected late [25]. This scenario is associated with more considerable deterioration in the quality of life, disabilities and higher mortality [9].

In 2005, the WHO recommended strategies for managing a flu pandemic threat, including specifying actions that countries and the international community should adopt to mitigate its impact [26]. Then, in a prescient paper in 2006, Battershill described potential problems for cancer patients emphasising in their susceptibility to infection and complications; moreover, he predicted the difficulties of access to health services associated with the lack of human resources in health and high costs [27]. Currently, from the observation, cancer patients are in a similar scenario dealing with the COVID-19 pandemic. This situation prompted us to conduct the analysis.

## Methodology

We carried out a multidisciplinary analysis from several perspectives of professionals working in cancer embracing surgical oncology, cancer medicine, radiotherapy, anaesthesiology, palliative care, cancer pathology, anthropology and epidemiology. The research team included specialists from the health sector—public and private—and academia. A literature review and lessons learned from healthcare experiences were the principal basis for its development. We used an Ishikawa diagram to identify the possible factors that affect disease progression and cancer death in the context of the COVID-19 epidemic. We organise various meetings via online platforms and generating records using Microsoft Word® (Microsoft Corporation, CA, USA).

## Analysis

**Figure 2.**
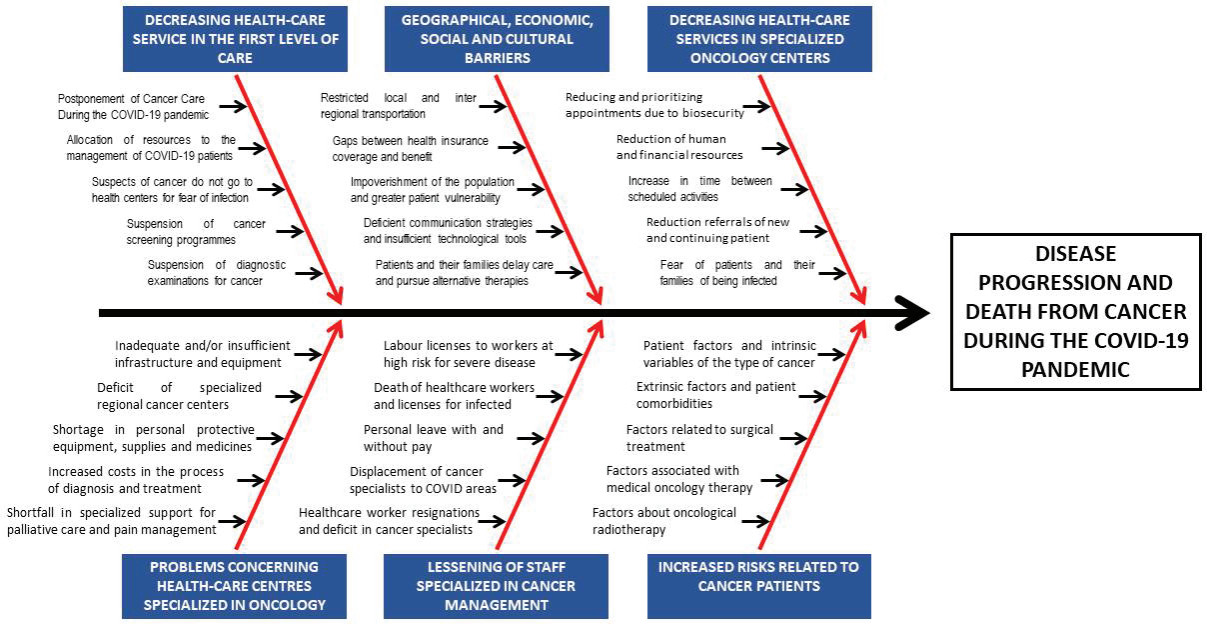
Ishikawa diagram about possible factors that affect disease progression and cancer death during the COVID-19 pandemic in Peru.

We identified a set of six areas that could affect—directly or indirectly—on the disease progression and death. Figure 2 shows the Ishikawa diagram performed.

## Decreasing services and facilities in primary healthcare and the first level of care

Primary healthcare is the basis of the health system in Peru; specifically, the first level of healthcare is usually the gateway to the PHS and is made up of health facilities that carry out low complexity care activities and preventive actions in the community. More recently, the range of healthcare activity has been modified and expanded, including health promotion, risk prevention and monitoring health problems, early diagnosis, timely treatment, rehabilitation and palliative care [28]. Nevertheless, ambulatory services at all health centres were restricted early in the pandemic.

### Postponement of Cancer Care During the COVID-19 pandemic

From the first confirmed COVID-19 case and the first death—reported on March 6th and 19th, respectively—and from the beginning of the national emergency and lockdown, most resources have been focused on preventing and managing SARS-Cov-2 virus infection. Consequently, the management of other public health problems, including cancer, has been postponed [29].

### Allocation of resources to the management of COVID-19 patients

Peruvian government performed projections about infections and deaths based on exponential growth curves from other countries. Thus, estimations served as support to assign the human and material resources, most for the implementation of strategies related to the detection and management of suspected and confirmed COVID-19 cases.

### People with suspected cancer fear becoming infected and have not gone to health centres

Demand for medical attention has decreased in these patients and their immediate relatives. Similarly, there is a marked reduction in screening evaluations. Some patients even hide or minimise their symptoms, postponing the possibility of timely diagnosis and referral to a specialised centre to receive curative treatment.

### Suspension of cancer screening programmes

The deferral of cancer triage and screening programmes during the pandemic led to more cancers at various stages being undetected. This particular situation is worrying in Peru, where a high proportion of cancer cases is diagnosed in the advanced and locally advanced stages [25]. Several asymptomatic patients will become symptomatic, and many will need palliative treatment.

### Suspension of diagnostic examinations for cancer

At the national level, several health centres conducted diagnostic procedures for cancer, such as tumoural markers, radiology, endoscopies and biopsies. Nonetheless, currently, the vast majority of these diagnostic resources is used in COVID-19 patients.

## Geographic, economic, social and cultural barriers

The supply of specialised centres for cancer treatment is still insufficient across the country. Cancer institutes provide specialised care to patients referred from other areas, where specialised human resources are scarce, and there is no logistical support to provide adequate oncological management [10]. INEN, in the capital city, is the leading national cancer centre, and roughly 50% of its patients come from other regions [30]. The experience of the regional institutes in Trujillo and Arequipa is similar, with 30%–40% of patients coming from other provinces [31, 32].

### Restricted local and inter-regional transportation

Travel represents a risk of contagion from COVID-19 and other infectious diseases. The state of emergency declaration restricted interprovincial transport by land, air, sea and river [29]. Populations generally have difficulty moving within their cities or towns, and obtaining a transit permit requires several administrative steps. However, in cancer patients, postponing travel could imply increasing the risk of disease progression.

### Gaps between health insurance coverage and access to health services:

The promulgation of the Universal Health Insurance Act in 2009 and the implementation of health sector reforms in 2013 foster a notorious expanding in health insurance coverage, with a 6.7% average annual increase from 2009 to 2017 [21]. Besides, based on the National Household Survey (ENAHO, from Spanish Acronym), in the third trimester of 2019, only 75.5% of the population had at least one health insurance, whereas the National Health Authority (SUSALUD from Spanish Acronym) reported in 88.1% in September 2019 [33]. The remaining population, when faced with cancer, will try to join the Comprehensive Health Insurance (SIS), a subsidised regimen, or they will be cared for in private institutions with a high out-of-pocket expense that could be catastrophic.

The net expansion in insurance coverage is not sufficient; it is necessary to reduce the gaps with access to health services. Several proposals have been addressed at mitigating the factors that restrict access to health services: lack in infrastructure and equipment, weak articulation in the first level of care, poorly paid human resources and absence of incentives, amongst others [22–24].

### Impoverishment of the population and greater patient vulnerability

Social immobilisation measures have caused the loss of income in a significant proportion of the economically active population, especially independent and non-formal workers. Some formal dependent employees have gone into the ‘perfect suspension of work’, thereby maintaining the employment relationship although without receiving any salary.

Besides this, families with a cancer patient experience a shortfall in their finances and assets during the diagnosis and treatment. Cancer, combined with poverty and psychological factors, causes deterioration in the general and nutritional state of patients, affecting their vulnerable immune status, an essential pillar in their response to treatment.

### Deficient communication strategies and insufficient technological tools

In 2017, the Telehealth Act was promulgated and subsequently regulated [34]. However, despite this current law, telehealth tools were not used regularly for almost 2 months from the beginning of lockdown. Recently, amongst the pandemic, the promulgation of Legislative Decree 1490 permitted the teleconsultation, telementoring and electronic prescribing, amongst other actions [35]. This strategy is a significant step that could help narrow the gap in access to medical care for patients throughout the country, following current health recommendations and guidelines [36–39].

Currently, consultations and interconsultations are carried out by telephone, where specialist physicians attend to patients and resolve doubts from other healthcare professionals, respectively. The ignorance of patients about these alternatives, the lack of technological tools and the absence of knowledge regarding their use are enormous barriers to telehealth implementation, especially in the most remote areas of the country. Another enormous problem is insufficient access to prescribed drugs. This trouble is associated with the healthcare provider, age and poverty, amongst other factors. In the current context, there are deficiencies in strategies that optimise drug delivery to patients who live far away from cancer care centres [23].

### Patients and their families postpone medical appointments and pursue alternative therapies

In some cases, cancer patients and their families opt for alternative therapies without scientific evidence. Regarding this, there are multiple associated factors, amongst them: fear of going to healthcare centres, difficulties in obtaining medical attention, high costs of treatments in private institutions, refusal to accept treatment and popular idiosyncrasy, amongst others. These therapies impoverish them and do not change the natural history of the disease. Another problem is cancer management by non-specialised professionals, who provide non-protocolised treatments without clinical benefits.

## Decreasing appointments and healthcare services in specialised oncology centres

In the case of specialised cancer centres, similar to the first healthcare level, initially, regular care was suspended until safe strategies for continuous care were adopted. The increasing number of cancer patients and the still insufficient supply of healthcare show that the pandemic has exacerbated the problems in providing cancer services.

### Reducing and prioritising appointments due to biosecurity

The declaration of the national state of emergency in March stopped all care activities in healthcare facilities, including oncology [29]. Only emergency area, hospitalisation and some patients undergoing treatments were kept. All face-to-face outpatient medical care was suspended in all areas; similarly, diagnostic and therapeutic processes were stratified and prioritised according to the type of cancer, prognosis and risk of progression.

### Reduction of human and financial resources

The care and administrative personnel from the different hospital areas decreased, mainly because of the categorisation into risk groups or vulnerable populations for severe COVID-19, in compliance with the health standard established by the government [40, 41]. Furthermore, financial resources initially allocated for the prevention, diagnosis and treatment of cancer was partially reallocated to cover other expenses related to COVID-19.

### Increase in time between scheduled activities

The reduction in the number of workers and the application of biosecurity measures—such as the careful disinfection of environments, equipment and instruments—have caused a more extended time between healthcare activities. This prolongation affected patient evaluations, diagnostic tests, surgical procedures and starting treatments. The longer time required between healthcare activities means fewer of them.

### Reduction referrals of new and continuing patients

Hospitals have directed their efforts into managing COVID-19 patients, and some are even exclusively dedicated to this purpose, whereas primary care facilities were initially closed. Furthermore, this stopped the process of detecting new cases of cancer and referrals of high suspicion and diagnoses of patients. Thus, in certain areas at specialised levels, a decreased demand for new and continuing patients is reported.

### Cancer patients and their families do not attend healthcare centres for the fear of becoming infected

The information, not necessarily valid and reliable, about SARS-CoV-2 infection has had an impact on the behaviour population. Besides this, the communication and circulating information about the lack of in-hospital support for infected patients, the uncertainty regarding treatment and the increase in infections and deaths have together caused a fear in patients and their family members, whereby they then do not attend specialised centres.

## Problems concerning healthcare centres specialised in oncology

Pre-pandemic projections estimated that the incidence of cancer would be higher in low- and middle-income countries in the subsequent decade [13, 14]. However, the COVID-19 pandemic has likely accelerated the presentation of this scenario in a shorter term. This situation could bring a demand that would exceed the problem-solving capacity in oncological health, reaching critical levels with increasing cases, disease progression and death.

### Inadequate and insufficient infrastructure and equipment

The extremely high demand for cancer patients in the country and the decentralisation of healthcare led to the initiation of activities in two specialised regional cancer centres: IREN North and IREN South just over a decade ago [11]. During this period, these institutes have carried out promotion, prevention, diagnostic and treatment activities. However, the limited infrastructure, deficits in equipment and lack of specialised personnel have caused treatment deferrals, and consequently, the transfer of patients to Lima, the capital city, persists [30]. Furthermore, in these regional institutes, medical offices, hospitalisation, the operating room and recovery are small areas, which, added to the fragility of the construction materials, increases the probability of contagion in health workers and patients.

### Deficiency of specialised regional centres

As previously described, Peru suffers a notorious deficit of dedicated cancer healthcare centres and specialised personnel in several cancer-related areas. One example is the delay in the construction of the new IREN North, whereas this institute continues working in an adapted infrastructure. In addition, IREN northeast planned in Iquitos has not yet started activities even though the laying of the first stone was more than 5 years ago. In 2017, the metropolitan area (Lima and Callao) concentrated most of the cancer specialists in their different subspecialties: surgical oncology (79.2%), clinical oncology (73%) and radiotherapy (82.6%) [42].

Various factors such as the shortage of specialists in local oncology units and deficiencies in logistical support for diagnosis and treatment in regional governments contribute to referring patients to IRENs, where the demand exceeds the offer of specialised services. This situation will probably worsen in the next few months, thereby postponing patients with advanced disease in favour of those who are potentially treatable with curative intent. Moreover, social security (EsSalud) is not far from this reality, with a higher average in waiting times for referrals, appointments and procedures.

### Shortage in personal protective equipment (PPE), supplies and medicines

The increase in the worldwide demand for various medical supplies—especially the PPE needed to continue working in hospital environments and offering care to patients—has caused scarcity and increased prices. Another core problem is the supply and distribution of drugs; this deficit will not allow the start or continuation of treatment, triggering disease progression and increased deaths. Similarly, there are hoarding, speculation and malicious activities that are not yet subject to criminal sanction in the country.

### Increased costs in diagnosis and treatment

The succession and interrelation of the various factors analysed in the previous sections contribute to increasing costs of the processes related to cancer care: transfer, diagnosis and treatment, amongst others.

### Shortfall in specialised support for palliative care and pain management

In the current context, many patients in units or functional teams for palliative care (PC) and oncological pain therapy have stopped attending and did not continue receiving treatment. They seek to alleviate the symptoms of cancer, and in several cases, they pursue support and accompaniment for a dignified death. These circumstances can impair the deteriorated health status in the majority of patients with advanced disease and probably have negative repercussions on their fragile homeostasis and maintenance of life. In response to this situation, the healthcare centres that provide PC services have implemented or optimised domiciliary care systems using telehealth [34, 35], an approach still underused in the health system. On the other hand, palliative medicine usually includes substances subjected to control by health authorities, such as narcotics and psychotropic drugs. For their acquisition, patients required a prescription with individual formats [43]. Therefore, institutions have established systems and logistic mechanism to provide the direct transport of drugs from pharmacies to the patient´s home; however, there are difficulties involved in its implementation since it depends on the resources and supply available in low complexity healthcare facilities, where frequently these medications are not available.

## Decrease of staff specialised in cancer management

The government established measures for the surveillance, prevention and control of the health of workers with the risk of exposure to COVID-19 [40]; however, a high percentage of healthcare personnel had sick leave, others became infected and some died.

The country, with almost 300,000 positive cases, 10,045 deaths, and an estimated average case fatality rate of 3.44% according to the MINSA reports at Julio 1 [44], has rapidly climbed positions to reach seventh place worldwide and second in the region after from Brazil in confirmed COVID-19 cases. This situation has occurred despite the fact that the Peruvian government has implemented—although with questionable adherence—several radical and aggressive political measures to mitigate the epidemic [5]. However, knowing the social and economic structural problems in Peruvian society is highly relevant to understand the population response and epidemic distribution.

### Labour licenses to workers at high risk for severe COVID-19 disease

Intending to protect all vulnerable people, the Peruvian government has granted work leaves to occupational groups based on high risk for severe COVID-19 disease. A legal decree defined high-risk groups including age (over 60 years), hypertension, cardiovascular disease, cancer, diabetes mellitus, obesity, asthma, chronic kidney disease and others conditions undergoing immunosuppressive treatments [40, 41].

### Leave of absence for people infected or suspected and deaths by COVID-19

Throughout the epidemic, SARS-COV-2 infection has reached health workers working in care and administrative areas, and given this, employers have granted them medical leave to receive treatment at home, general hospitalisation or critical care. However, a significant proportion of them have died, and some survivors have developed serious sequelae that prevent their return to the workplace. On July 1, 2,062 doctors have been infected, 71 died, and 53 are in critical care units; besides this, more than 1,300 nurses have tested positive for COVID-19 [45].

### Personal leave with or without pay

Some workers not classified in the high-risk occupational groups have applied for work leave without salary or have used their vacation period during the confinement. They pursue avoiding exposure to SARS-COV-2 under the precept that hospitals pose a high risk of infection.

### Displacement of staff specialised in cancer care to work in COVID-19 areas

The increase in the demand for care in exclusive COVID-19 areas and the decline in the number of healthcare workers—due to death, illness or other labour leaves—has caused the mobilisation of cancer specialised workforces to these areas. Although this strategy reinforces the COVID-19 control, it creates a deficit in cancer care.

### Healthcare worker resignations and deficit in cancer specialists

Some healthcare workers have resigned during the COVID-19 state of emergency. There are several factors associated with this desertion, including fear of disease and its complications, fear of death, concern about infecting family members, lack in personal protective equipment, type of employment contract, displacement to high-risk areas and job offers with better working conditions in other institutions.

## Increased risks related to cancer patients

The claim that ‘a cancer survivor’ inherently has a higher probability of SARS-CoV-2 infection compared to the general population, is controversial; there is no published scientific evidence to support this [46–49]. Instead, these patients go to hospitals and healthcare centres more frequently with an increase in the risk of contagion [36, 47–51].

Several studies have reported that cancer patients are at higher risk of severe COVID-19, many will need critical care and others will die. This situation is related to the immunosuppression due to their disease or treatment and other factors such as age and comorbidities (cardiac, chronic obstructive pulmonary disease, diabetes, obesity or smoking) [36, 47–52].

### Patient-related factors and intrinsic variables of the type of cancer

Scientific evidence has demonstrated that advanced age is associated with cancer, and preliminary communications report that age is an independent risk factor for mortality in COVID-19. The Chinese Centre for Disease Control and Prevention reported that, based on a series of 44,672 confirmed cases, the case fatality rate (CFR) in the general population was 2.3%, whereas CFR was 14.8% in 80 years and older [53, 54]. In Peru, official reports show 10,045 deaths (69%) corresponding to 60 years and older (July 2, 2020) [44]. Several factors could explain the effect of age: immunosenescence—the gradual deterioration of the immune system produced by natural age advancement–loss of general function, physical condition and increased frailty associated with ageing [55].

Within cancer patients, according to several publications, there are more vulnerable groups: haematologic neoplasms (more frequent myeloid than lymphoid neoplasms), metastatic diseases, pulmonary cancer, gastrointestinal (colon, pancreas and upper gastrointestinal tract), head and neck cancer and gynaecological neoplasms, patients who receive treatment with haematopoetic precursor cell transplants, patients with active disease and recent diagnosis (less than 1 year) and patients receiving immunotherapy (higher risk of acute respiratory distress syndrome) [29, 37, 56].

### Extrinsic factors and comorbidities

The lockdown has lowered overall family income and has likely adversely affected the nutritional status of cancer patients. Similarly, cancer patients who live in closed spaces such as retirement homes and those who live in overcrowded and poor sanitary conditions, such as in large prisons, will have a higher risk of infection. Comorbidities such as cardiovascular diseases, hypertension and diabetes mellitus increase the risk of severe COVID-19 disease, and this would be related to the overexpression of ACE-II receptors and the decrease in humoral and cellular immunity. Furthermore, some reports have described that chronic respiratory diseases and immunodeficiencies increase the probability of severe COVID-19 and death [40, 45, 46, 50, 54].

### Factors related to surgical treatment

The suspension of scheduled surgeries was an initial step during the pandemic. Currently, surgical interventions are performed in the following cases: emergencies, it is the only therapeutic alternative, or waiting time affects the clinical prognosis due to disease progression [29]. In cancer patients, the loss of timely therapy is catastrophic; the postponement of surgical treatment enhances the risk of disease progression and death. The potential complications in surgeries performed in the context of a pandemic are not yet known. Hence, publications report a case fatality rate of up to 20% in asymptomatic COVID-19 patients who were involuntarily scheduled for elective surgery [57]. Furthermore, a Chinese study reported that the prognosis of COVID-19 patients is worse in cancer patients who have undergone surgery in the past year [47]. The international surgical societies have circulated recommendations related to delaying elective procedures according to specialist evidence during the acute period of the pandemic and to minimising the use of necessary elements to care for infected patients such as critical care beds, personal protective equipment, cleaning supplies for terminals and ventilators [58]. The risk due to the lack of blood products and support in the perioperative period should also be considered in extensive cancer surgeries [59].

### Factors associated with medical oncology therapy

The decision to maintain or initiate systemic therapy requires an initial evaluation including the characteristics of cancer, the general condition of the patient, the potential benefit not achievable with other therapies and the risks of complication in a hypothetical infection scenario [36, 39, 50, 51].

The delay or lack of treatment would lead to disease progressions, clinical deterioration and patient functioning with the subsequent loss of opportunity of a treatment that may improve their survival and quality of life and occasionally have curative intent. Therefore, although there are recommendations from several institutions and organisations [36–39], making decisions about a specific case is complex and needs a multidisciplinary team analysis.

Consequently, there is a high likelihood of infection and complications in cancer patients during the diagnostic or therapeutic process. In China, a study on cancer patients and COVID-19 reported that admission to critical care for severe respiratory complications was higher in people who received chemotherapy in the past month [47].

### Factors about oncological radiotherapy

In the current situation, where surgery and chemotherapy offer increased risks for the patient and health personnel, radiotherapy has become the local treatment option for many types of cancer. In recent months, different scientific societies have published standards for the treatments of several oncological pathologies. In them, the biosecurity of the patient and healthcare personnel is a priority; also, the treatment is prioritised for the types of cancer with the highest risk.

At INEN, the Department of Radiotherapy, throughout the quarantine, has provided uninterrupted care. The measures implemented are the triage and search for asymptomatic patients, the provision of personal protective equipment to the staff and disinfection of the treatment rooms. Similarly, to prevent over-exposing patients to SARS-COV-2 infection, the department avoided giving treatments to people who could benefit from other modalities; furthermore, it was averted to defer therapies to post-lockdown periods, and radiotherapy schedules were shortened in cases requiring immediate treatment.

For several years, there has been clinical evidence to apply high doses of radiation in a short time, the techniques of moderate hypofractionation (total treatment time less than 4 weeks compared to conventional fractionation over up to 8 weeks) and ultra-hypofractionation in 5 days maximum. The implementation of these techniques implies fewer visits to the radiotherapy centre and reduces the risks of exposure for patients and their families and healthcare staff. On the other hand, the evidence shows that the suboptimal administration of radiotherapy (delay, interruption or omission) compromises local control and survival [60].

## Conclusion

In more than 120 days after the declaration of a state of health emergency due to COVID-19 in Peru, and from the analysis performed, there is indirect evidence that suggests a potential increase in morbidity, disease progression and mortality related to cancer. Although we did not show empirical evidence, due to the difficulty of access to administrative records and other data sources, this approach would indicate excess in these three outcomes compared to previous years.

In this context, it is appropriate to carry out well-designed studies to obtain valid and reliable estimates of the impact of the COVID-19 pandemic on the progression of disease and death of cancer patients in Peru. The ‘excess mortality’ is an important indicator, from which it is possible to classify deaths due to the direct effect of SARS-CoV-2 infection, and those produced by the indirect effects of the epidemic, such as the case of patients without timely and adequate diagnosis and treatment. Consequently, cancer control emerges as a daunting mission; thus, cancer research must be prioritised in the national agendas.

## Conflicts of interest

The authors declare that they have no conflicts of interest.

## Funding

None.
